# StrainR2 accurately deconvolutes strain-level abundances in synthetic microbial communities

**DOI:** 10.1093/bioinformatics/btaf440

**Published:** 2025-08-06

**Authors:** Kerim Heber, Shuchang Tian, Daniela Betancurt-Anzola, Heejung Koo, Jordan E Bisanz

**Affiliations:** Department of Biochemistry and Molecular Biology, Pennsylvania State University, University Park, PA 16802, United States; Department of Biochemistry and Molecular Biology, Pennsylvania State University, University Park, PA 16802, United States; Department of Biochemistry and Molecular Biology, Pennsylvania State University, University Park, PA 16802, United States; Department of Biochemistry and Molecular Biology, Pennsylvania State University, University Park, PA 16802, United States; Department of Biochemistry and Molecular Biology, Pennsylvania State University, University Park, PA 16802, United States; One Health Microbiome Center, Huck Life Sciences Institute, University Park, PA 16802, United States

## Abstract

**Motivation:**

Synthetic microbial communities offer an opportunity to conduct reductionist research in tractable model systems. However, deriving abundances of highly related strains within these communities is currently unreliable. 16S rRNA gene sequencing does not resolve abundance at the strain level and other methods such as quantitative polymerase chain reaction (qPCR) scale poorly and are resource prohibitive for complex communities. We present StrainR2, which utilizes shotgun metagenomic sequencing to provide high accuracy strain-level abundances for all members of a synthetic community, provided their genomes.

**Results:**

Both *in silico*, and using sequencing data derived from gnotobiotic mice colonized with a synthetic fecal microbiota, StrainR2 resolves strain abundances with greater accuracy and efficiency than other tools utilizing shotgun metagenomic sequencing reads. We demonstrate that StrainR2’s accuracy is comparable to that of qPCR on a subset of strains resolved using absolute quantification.

**Availability and implementation:**

Software is available at GitHub and implemented in C, R, and Bash. Software is supported on Linux and MacOS, with packages available on Bioconda or as a Docker container. The source code at the time of publication is also available on figshare at the doi: 10.6084/m9.figshare.29420780.

## 1 Introduction

Most metagenomic tools are unable to quantitatively resolve strain-level abundances; however, variation at the strain level is a crucial determinant of microbiome function and host–microbe interactions (Carrow *et al.* 2020, Van Rossum *et al.* 2020, Anderson and Bisanz 2023). As a well-known representative, some *Escherichia coli* strains are pathogens causing severe diarrhea, while others are described as probiotics used in treating diarrhea (Leimbach *et al.* 2013). Strains of the same species (clonal populations within the species often represented by a cultured isolate) share a small proportion of their genome referred to as the core genome. However, many of the genes that drive important phenotypes are found in the variable, or accessory, portion of the genome (Medini *et al.* 2005). Understanding the role that intra-species (infraspecific) variation plays in microbiome function and the competitive interactions within these species is crucial for advancing our knowledge of how complex microbial communities assemble and function.

Synthetic communities offer a powerful tool that balances experimental reductionism with a biologically relevant scale across multiple systems (Becker *et al.* 2011, Grosskopf and Soyer 2014, Vázquez-Castellanos *et al.* 2019, Charbonneau *et al.* 2020, Cheng *et al.* 2022, Wang *et al.* 2023, van Leeuwen *et al.* 2023, Aguilar-Salinas and Olmedo-Álvarez 2023). These synthetic communities are providing important insights into the assembly and function of microbial communities across a range of ecosystems and applications including crop health (Knoth *et al.* 2014, Durán *et al.* 2018, Carrión *et al.* 2019, Coker *et al.* 2022, Gonçalves *et al.* 2023, Ma *et al.* 2024), marine systems (Fu *et al.* 2020), and the intestinal microbiota (Woting *et al.* 2014, 2015, Slezak *et al.* 2014, Wolf *et al.* 2019, Krause *et al.* 2020, Cheng *et al.* 2022, Nagashima *et al.* 2023). They are providing important answers to biological questions on the role of microbes including in physiological development (Slezak *et al.* 2014), aging (Perez *et al.* 2021), immune function (Atarashi *et al.* 2011, 2013, Tanoue *et al.* 2019, Nagashima *et al.* 2023), response to drugs (Garcia-Santamarina *et al.* 2024), synthetic fecal microbiota transplant (FMT) (Petrof *et al.* 2013, Tian *et al.* 2025, Fishbein *et al.* 2025), and human diseases (Lengfelder *et al.* 2019, van der Lelie *et al.* 2021). An important component of these experiments is understanding how abundant and prevalent the strains within the synthetic communities are. While this is highly challenging in undefined communities, a unique consideration of synthetic communities is that they are normally constructed from genome-sequenced constituents, which provides a constrained reference for the set of reads that may arise from metagenomic sequencing.

**Figure 1.**
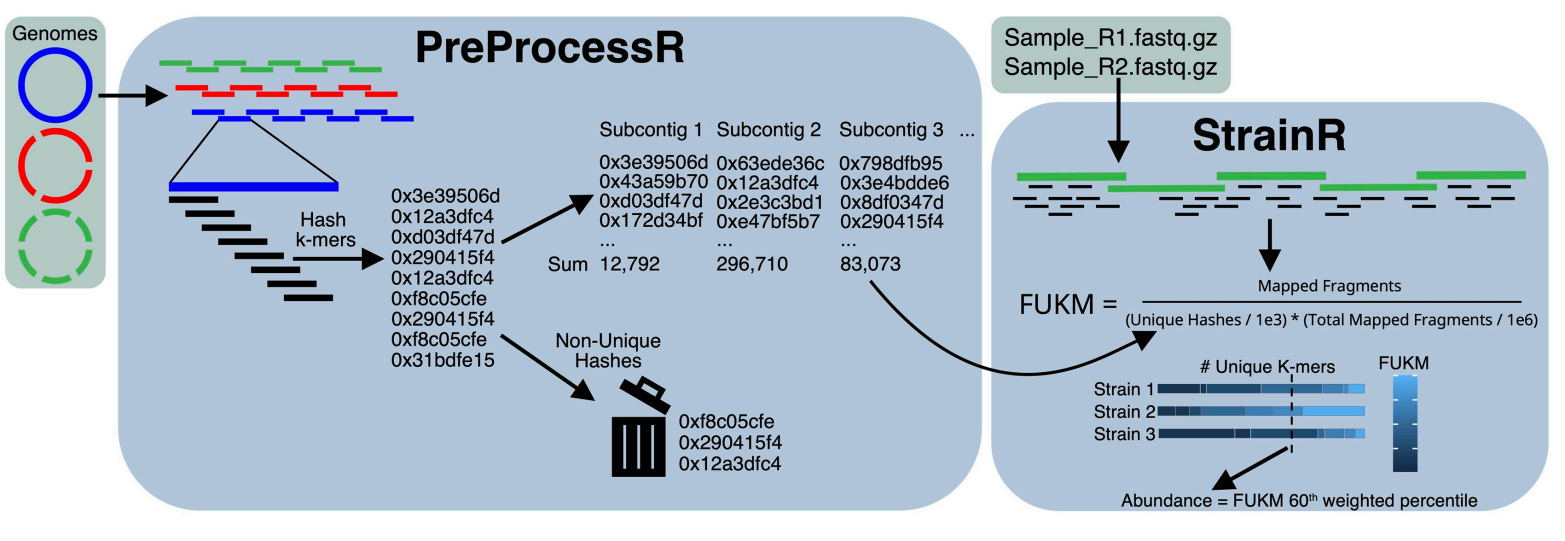
Schematic of the StrainR2 workflow. In the PreProcessR module, genomes are split into subcontigs no larger than the smallest N50 in the set of genomes ensuring consistent assembly qualities. The number of unique k-mers (which are computed as hashes for efficiency) is used in the StrainR module to normalize FPKM in a metric normalized for genome uniqueness (FUKM). A user-configurable weighted percentile of all subcontig FUKMs belonging to a genome is used as a point estimate of abundance.

In complex communities, culture-based methods of quantification including selective/differential media are often insufficient to resolve all strain members. Strain-specific quantification via qPCR represents a gold standard; however, the cost and time associated with assay development, validation, and sample processing render this approach non-cost competitive when applied to more complex communities. Traditional sequencing-based methods for quantifying organism abundances, such as 16S rRNA gene sequencing, usually lack the resolution to differentiate strains and are limited to generalizing to the species, genus, or higher taxonomic levels as a function of divergence within the clade of interest (Johnson *et al.* 2019) (Fig. 1A, available as supplementary data at *Bioinformatics* online). Much sequencing data, including shotgun metagenomic sequencing, is often quantified using the metric mapped fragments per kilobase per million reads (FPKM). The limitation of this approach for strain-resolved abundances is how to deal with reads that map to multiple strains, or ambiguous reads. Partially or randomly assigning ambiguous reads to all genomes that they map to introduces noise and inflates the abundance of low-abundance and/or absent strains. Ignoring these reads, on the other hand, may lead to a bias where genomes that are more similar to each other have artificially reduced observed abundances. As such, NinjaMap (Cheng *et al.* 2022) and StrainR (Bisanz *et al.* 2020), here referred to as StrainR1, were developed to address these challenges in a 119-member community of diverse gut microbes, and a 22-member community of entirely *Eggerthella lenta* strains (Bisanz *et al.* 2020), respectively. Both approaches explicitly include a normalization strategy to correct for the uniqueness of the target genome; though they differ significantly in their implementations. In addition, StrainScan (Liao *et al.* 2023) was also developed to optimize stain-level analysis in metagenomic sequencing reads using an approach of clustering strains and interpreting k-mers in their relation to these clusters, though our testing indicates it scales poorly to large synthetic communities and demonstrates low accuracy for low-abundance strains.

NinjaMap tackles the problem by using the proportion of uniquely mapped reads to partially assign ambiguously mapped reads to optimize the use of all the reads available from a sequencing run. In addition, reads are generated *in silico* as part of the pipeline to assess and normalize for strain uniqueness. This does not necessarily solve the bias observed from uniquely mapped reads preferring more unique genomes, as the bias may be propagated to the assignment of ambiguously mapped reads. NinjaMap, therefore, exhibits some of the same problems as data that has not been normalized for unique mapping sites, as demonstrated in our analyses. Furthermore, this approach may lead to high false positive rates as it becomes likely that a genome has some number of uniquely mapped reads through either sequencing error, trace cross-contamination with input communities, or index hopping/barcode switching (Costello *et al.* 2018). Another problem faced by NinjaMap and StrainR1 is that they scale non-linearly, requiring extensive resources for complex communities that quickly run out of the resources required to run for analysis of large communities. Unlike NinjaMap, StrainR1 aims to only use uniquely mapped reads and normalize the bias toward more unique genomes directly by using the number of unique k-mers within each genome. This conceptually intuitive approach equates to reporting FPKM for highly unique strains while offering an effective normalization strategy for those that have closely related strains present. Unfortunately, the implementation of StrainR1 fell short of being able to scale to larger and more diverse communities requiring excessive computational resources, and it was not extensively experimentally validated. A third approach (COPRO-Seq) has been developed and applied to these problems (McNulty *et al.* 2011, Wolf *et al.* 2019); however, it is bespoke in its implementation and not supported for external researchers. This presents a need for a publicly available tool that can scale to run in most computing environments to enable the analysis of complex synthetic communities while still maintaining the same, or better, accuracy.

More general approaches for taxonomic identification of metagenomic data, such as MetaPhlAn 4 (Blanco-Míguez *et al.* 2023), are not appropriate for strain-level analyses due to their database construction and inability to resolve strains, similar to 16S rRNA sequencing (Fig. 1B and C, available as supplementary data at *Bioinformatics* online). Tools such as KMCP (Shen *et al.* 2023) and Kraken2 (Wood *et al.* 2019) also fall short in cases where not all strains of a community have a unique NCBI taxonomy ID and are, therefore, unable to be resolved without significant effort to rederive reference databases. Other tools for consideration include Strainer (Aggarwala *et al.* 2021), which was designed to detect the presence or absence of strains implanted into an undefined community using FMTs; however, it is not appropriate for use in the analysis of synthetic communities due to its intended application in undefined communities to find informative k-mers against a background community. Another alternative for determining the presence or absence of strains that may be applied to synthetic communities is YACHT (Koslicki *et al.* 2024): a tool that determines strain presence based on metagenomic sequencing reads. Neither of these approaches is designed for quantitative analysis of strains, but they can still be applied to benchmark the presence/absence detection of StrainR2.

In this article, we introduce, benchmark, and experimentally validate StrainR2, which quantifies strain abundances to a higher degree of accuracy than other methods relying on shotgun metagenomic sequencing data while maintaining scalable and lightweight run times and resource usage. StrainR2 can run both in a high-performance computing environment and on a personal computer. In our analyses, we find that many currently available tools derive inaccurate abundances, particularly for cases of low-abundance organisms or communities that contain highly similar strains. We further validate the superior accuracy of StrainR2 by benchmarking against samples derived from synthetic community colonized gnotobiotic animals characterized with strain-specific qPCR. StrainR2 will facilitate synthetic microbial community research of increasing complexity as it scales to study complex host-associated microbiomes like the mammalian gut.

## 2 Materials and methods

StrainR2 has two steps: (i) preprocessing (PreProcessR) and (ii) normalization (StrainR, Fig. 1). In short, PreProcessR first generates a reference database of normalization factors to be applied to each genome. This step needs only be run once per reference synthetic community. Next, StrainR uniquely maps reads on a per-sample basis to reference genomes and leverages the normalization factors in a modified FPKM formula termed fragments per thousand unique k-mers per million reads mapped (FUKM). A user-configurable weighted percentile of all subcontig FUKMs in a genome is used as the final measure of abundance. FUKM is directly analogous to FPKM, with the difference of normalizing the uniquely mappable sites of the genome rather than the total genome size.

### 2.1 Preprocessing

The preprocessing step is almost entirely written in C to maximize efficiency. It starts by splitting contigs of all genomes into similarly sized pieces (subcontigs) to ensure the build quality for all genomes is comparable. This eliminates bias toward build quality and also ensures there are sufficient subcontigs for use in the normalization step by providing multiple estimates of strain abundances and thereby normalizing out the effects of highly unique multi-copy elements such as plasmids and transposons. By default, the smallest N50 build quality for all provided genomes is used as the maximum possible size of subcontigs. StrainR2 maximizes subcontig size given the constraint of the smallest N50 being the upper bound. Subcontigs also have a 500 base overlap with the preceding and proceeding subcontigs to ensure reads in between subcontigs are mapped. Contigs under a minimum size, set to 10 kb by default, are excluded from the final calculation for FUKM but will still have their k-mers marked as non-unique. These small contigs could represent multi-copy elements and therefore bias read mappings if their k-mers are considered unique.

Canonical k-mers are generated and represented as 64-bit hashes for the sake of computational speed. The k-mer size is set to the paired-end read size plus one so that k-mers are canonical and each unique k-mer is analogous to a potential mapping site for uniquely mapped reads. In our internal testing, we found that using a read-sized k-mer size provided the most accurate results, as smaller k-mers under-normalized highly similar strains.

The non-cryptographic MurMurHash is used as the hashing function due to its speed. Unique hashes (or k-mers) in a subcontig are measured as being unique with respect to all genomes in the community. To determine which k-mers are unique in each subcontig, StrainR2 iteratively adds each k-mer’s hash to a table of all hashes encountered in the community thus far. As this is done, a count for the number of unique hashes in each subcontig is maintained, and counts are only correct given the k-mer hashes processed so far. This means that if a k-mer hash is encountered that was previously considered unique, it is marked as non-unique, and the unique k-mer count for its respective subcontig is decremented by one. The hash table is implemented with open addressing and a linear probe collision policy, as hash table entries are relatively small and can take advantage of spatial locality. The hash table is resized to powers of two whenever the load factor exceeds 0.75 after the hashes for a subcontig are added. One caveat to using hashes in place of k-mers is the possibility that two k-mers may have the same hash (collision). Assuming 150 base paired-end-reads are used (leading to the default use of 301-mers), that indicates that 1.7e181 possible k-mers will map to 1.8e19 possible hashes. 10 s or 100 s of millions of different k-mers, meaning the probability of different k-mers having the same hash is low and the effect is negligible.

### 2.2 Normalization

In the normalization step, reads are trimmed using fastp (Chen *et al.* 2018) with the options: trim_poly_g, length_required = 50, and n_base_limit = 0. BBMap is subsequently used to obtain the count of fragments that perfectly and unambiguously map to each subcontig, with the options: perfectmode=t, local=f, ambiguous=toss, pairedonly=t. Finally, FUKM is calculated using the formula:$\;\frac{\mathrm{Mapped}\;\mathrm{Fragments}}{\left(\mathrm{Unique}\;\mathrm{Hashes}/1e3)\;*\;(\mathrm{Total}\;\mathrm{Mapped}\;\mathrm{Fragments}/1e6\right)}$. To obtain a singular estimate for the abundance of a strain, two values are reported: the median FUKM across all of a strain’s subcontigs (mFUKM) as reported by StrainR1, and a user-configurable weighted percentile of subcontig FUKMs (wpFUKM). In brief, wpFUKM is calculated by sorting all of a strain’s subcontigs on the basis of their individual FUKM. The cumulative proportion of unique k-mers is then calculated along the sorted subcontigs, and the wpFUKM is returned as the FUKM of the subcontig that represented the user-definable x^th^ cumulative percentage of k-mers. Weighting by the number of unique k-mers allows for regions that are more representative of unique mapping sites to have more of an effect on the final abundance. Both the wpFUKM and the mFUKM results are reported in the output with the suggestion to carry wpFUKM forward in downstream analysis.

### 2.3 Unit testing

The stability and reproducibility of StrainR2 is validated by automated testing (GitHub workflow) to ensure that the output at each step of the workflow is reproducible and deterministic. Testing includes running PreProcessR and StrainR as shell commands in a conda environment, as well as running individual components of each command separately. For PreProcessR, this includes testing that genome contigs are correctly and completely divided into subcontigs, as well as ensuring that unique k-mer hash counts are correct. For StrainR, the calculation for FUKM is validated. This testing also ensures that StrainR2’s output remains consistent through updates to itself or its environment, as testing is triggered on any change to the source code.

### 2.4 *In silico* read generation

**Figure 2.**
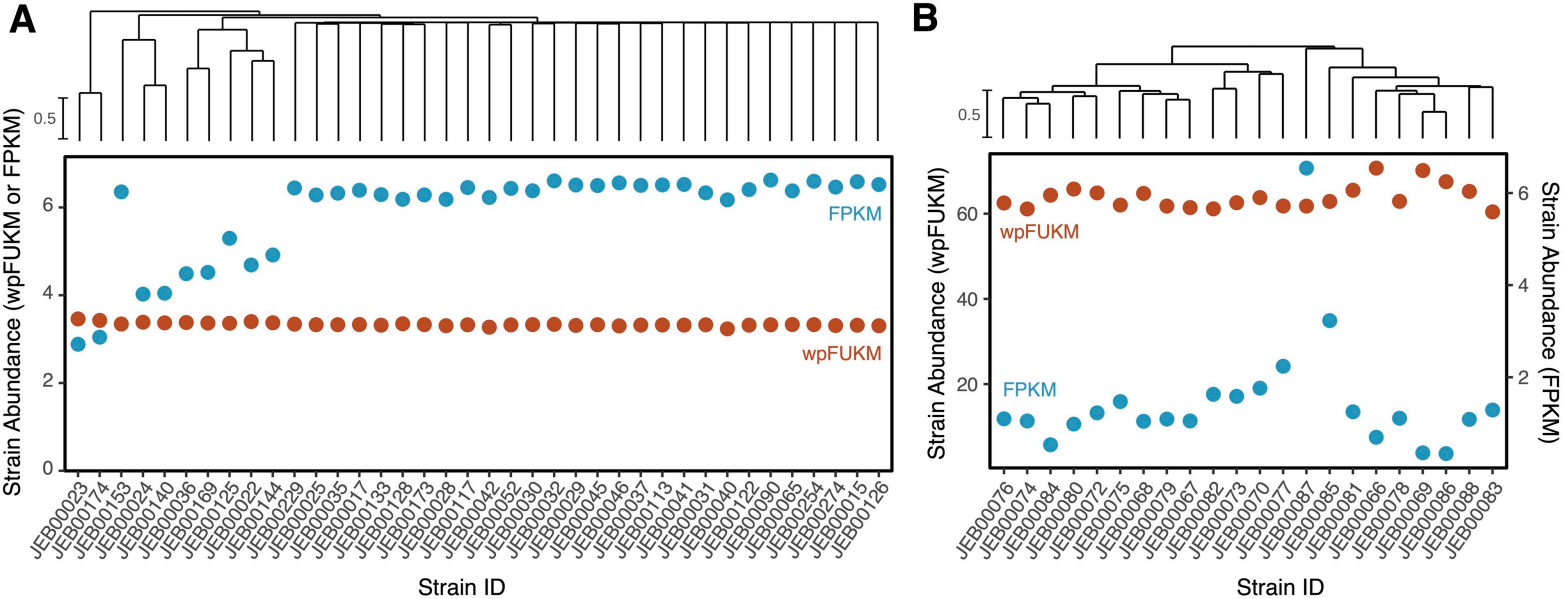
StrainR2 normalization corrects quantitative errors resulting from variable strain-relatedness. Dendrograms of strain similarities are shown for (A) sFMT1+Cs members and (B) 22 *E. lenta* strains. Reads were generated *in silico* such that all community members have a uniform abundance. StrainR2 resolves abundances much closer to the uniform abundance than by measuring FPKM. FPKM values for *E. lenta* strains were less accurate than with the sFMT1+Cs community due to an increased bias toward unique community members. StrainR2-calculated wpFUKM had a coefficient of variation of 1.69% for the sFMT1+Cs strains and 3.93% for the *E. lenta* strains despite the high strain similarity, whereas when using FPKM, the coefficient of variation was 17.44% and 86.82% for the communities, respectively. Dendrograms are based on Jaccard Similarity of k-mer profiles between strains.

For the purpose of assessing accuracy and speed, InSilicoSeq (Gourlé *et al.* 2019) was used to generate reads for the desired community and read depth. The coverage option was used with predetermined coverages to simulate these distributions, as well as the read depth option when reads with varying depths were desired (Fig. 2 and Tables 1–4, available as supplementary data at *Bioinformatics* online). Paired-end 150 bp reads from a NovaSeq 6000 S4 flow cell, which generated later experimental validation data, were used to create a custom error model for all *in silico* reads.

### 2.5 Method analyses

Unless otherwise noted, StrainR2 was always run with default options: a weighted percentile of 60, max subcontig size of the lowest N50 in all input genomes, minimum subcontig size of 10Kb, read size of 150, and no additional subcontig filtering. NinjaMap, MetaPhlAn 4, KMCP, Kraken2, and StrainScan were also run with default options.

Ambiguous strain resolution for 16S was decided by whether an amplicon sequence variant (ASV) from 16S rRNA sequencing data mapped to more than one strain, allowing for 1 mismatch. All strains that are mapped to by such an ASV are considered ambiguous. The *E. lenta* strains community did not contain any unique v4 region and was therefore considered to be completely ambiguous. Ambiguity for MetaPhlAn 4 was decided by whether a strain had a unique representative in the output by taxonomic name and aliases. For 3 strains in the *E. lenta* community, StrainScan was consistently unable to report the abundance of the strains, and they were considered ambiguously mapped. For all other tools, unique mapping was confirmed by an output that uniquely represents the input strain.

FPKM values were taken from the same mapping data that StrainR2 normalizes. More specifically, BBMap was run with the options: perfectmode=t, local=f, ambiguous=toss, pairedonly=t, and nodisk=t. All other options were left at the default. The FPKM was then calculated by summing the mapped fragments for each contig belonging to a strain in the .rpkm file and normalizing it by kilobases and millions of total reads.

To test StrainR2’s ability to determine strain presence/absence, it was benchmarked against YACHT (Koslicki *et al.* 2024). While it does not provide abundances, it can still be benchmarked against StrainR2 based on whether StrainR2 provides an abundance of 0 or not. YACHT was run with k-mers of size 31 with the scaled option set to 100. Significance was set to 0.99, and a minimum coverage of 0.01 was used.

### 2.6 Tool scalability analysis

Three hundred genomes were selected from an in-house collection of lab strains such that at least 2% of each strain’s 301-mers were unique. Reads were generated for random subsets of this community up to 300 genomes at a depth of 20 million reads, as well as for the full 300-genome set at varying read depths between 2 million and 20 million reads. The coverages for each strain were drawn from a log-normal distribution. Reads were also generated for the sFMT1+Cs community at depths between 250 000 reads and 2.5 million reads with a log-normal distribution.

### 2.7 Resource expenditure profiling

Tool preprocessing steps were tested on varying input sizes of random genomes belonging to human gut microbes from our in-house strain collection: between 10 and 200 genomes, as well as varying input sizes of the 22 *E. lenta* strains. Testing was done on an Ubuntu server with dual Intel Xeon Silver 4214 CPUs and 384 GB of memory. When resource usage for any tool became too large to be run with the computing resources available, or if the tool threw an error, testing was stopped. Where multithreading was an option, namely in the case of StrainScan, 16 threads were used.

### 2.8 Experimental validation

Mice (strain C57BL/6J) aged 8–17 weeks were given *ad libitum* Lab Diet 5021 and had a 12-hour light/dark cycle. Mice were housed inside Class Biologically Clean germ-free isolators in the gnotobiotic animal facility at Pennsylvania State University. Animal experiments were performed under an approved protocol (PSU IACUC PROTO202101826). Synthetic communities were assembled by pooling approximately equal cell densities of individual strains described elsewhere (Table 1, available as supplementary data at *Bioinformatics* online). Mouse fecal samples were extracted following the International Human Microbiome Consortium Protocol Q (https://human-microbiome.org/index.php?id=Sop&num=006). Briefly, samples were homogenized through bead disruption before isopropanol precipitation and extraction using the Qiagen Stool DNA kit. Libraries were prepared with the Illumina Library Preparation kit, and sequenced on a NovaSeq 6000 with a S4 flow cell (Novogene USA) to generate 150 bp paired-end reads at an average of 29.878 million reads per sample. Data is available for download at PRJNA1038784. Negative controls and fecal material from germ-free animals were also prepared and pooled for sequencing despite failing library construction QC. Reads from these communities were mapped primarily to the mouse genome and/or common reagent contaminants.

Strain abundances were quantified using qPCR for JEB00023, JEB00029, JEB00174, and JEB00254 (Table 1, available as supplementary data at *Bioinformatics* online). Primers were designed using PrimerBLAST to be specific to unique portions of each target genome. Primers were validated *in silico* and experimentally using gDNA from the pure culture members of the sFMT1+Cs community via qPCR to ensure a lack of cross-reactivity. The primers used are as follows: JEB00023 forward primer: GGCACTCATCGGAGGTTTCA, JEB00023 reverse primer: CGTTGGGCTTGTCACCAAAG JEB00029 forward primer: TCATGGCCGTGTACTTGCTT, JEB00029 reverse primer: AGCGGATATCTGCCAGGTTG, JEB00174 forward primer: TGGAGTTCGGCGTAGCTTTT, JEB00174 reverse primer: TCTCGGCATTCCAACCAGAC JEB00254 forward primer: ACAGGCTTTGGCATTGGAGA, and JEB00254 reverse primer: TGTGGTTAATGGCCTTGCAT, with the concentration of each being 200 nM. Samples were amplified with iTaq Universal SYBR Green Supermix (Biorad 1725122) at 95°C for 3 min, followed by 40 cycles of 5 s at 95°C and 30 s at 60°C using a Biorad CFX384 Opus. Strains were quantified against standard curves of pure gDNA and normalized by extracted sample mass to calculate absolute abundance.

## 3 Results

Throughout the analysis, two communities of strains were used to generate reads *in silico* across 6 abundance distributions that represent various scenarios (Fig. 2 and Tables 1–4, available as supplementary data at *Bioinformatics* online). The first community, sFMT1+Cs, represents a more realistic scenario for a synthetic community where most strains are not very similar, but five of the species have two or more strains each (Fig. 2A, Table 1, available as supplementary data at *Bioinformatics* online). As in the development of StrainR1, a mock community of 22 *E. lenta* strains was also used to represent an extreme scenario where the number of unique k-mers would be at a minimum (Fig. 2B).

To evaluate StrainR2’s improvement over using FPKM values in the case of uniformly abundant strains, the coefficient of variation was used as a benchmark, as lower coefficients of variation are closer to a uniform distribution. In the case of the sFMT1+Cs community with uniformly abundant strains, StrainR2’s wpFUKM was able to normalize the reads such that the coefficient of variation was 1.69% as compared to FPKM with a coefficient of variation of 17.44% (Fig. 2A). In the case of uniformly abundant *E. lenta* strains, the coefficients of variation were 3.93% and 86.82% for wpFUKM and FPKM, respectively (Fig. 2B). Using StrainR2, this corresponds to fold-change differences of 1.085 and 1.159 between the highest and lowest reported abundances for wpFUKM on sFMT1+Cs and *E. lenta* strains, respectively. Despite the high similarity between strains, wpFUKM still resembled a uniform distribution, unlike FPKM. FPKM tended to underestimate the abundance of strains with higher similarity, whereas wpFUKM remained unbiased.

**Figure 3.**
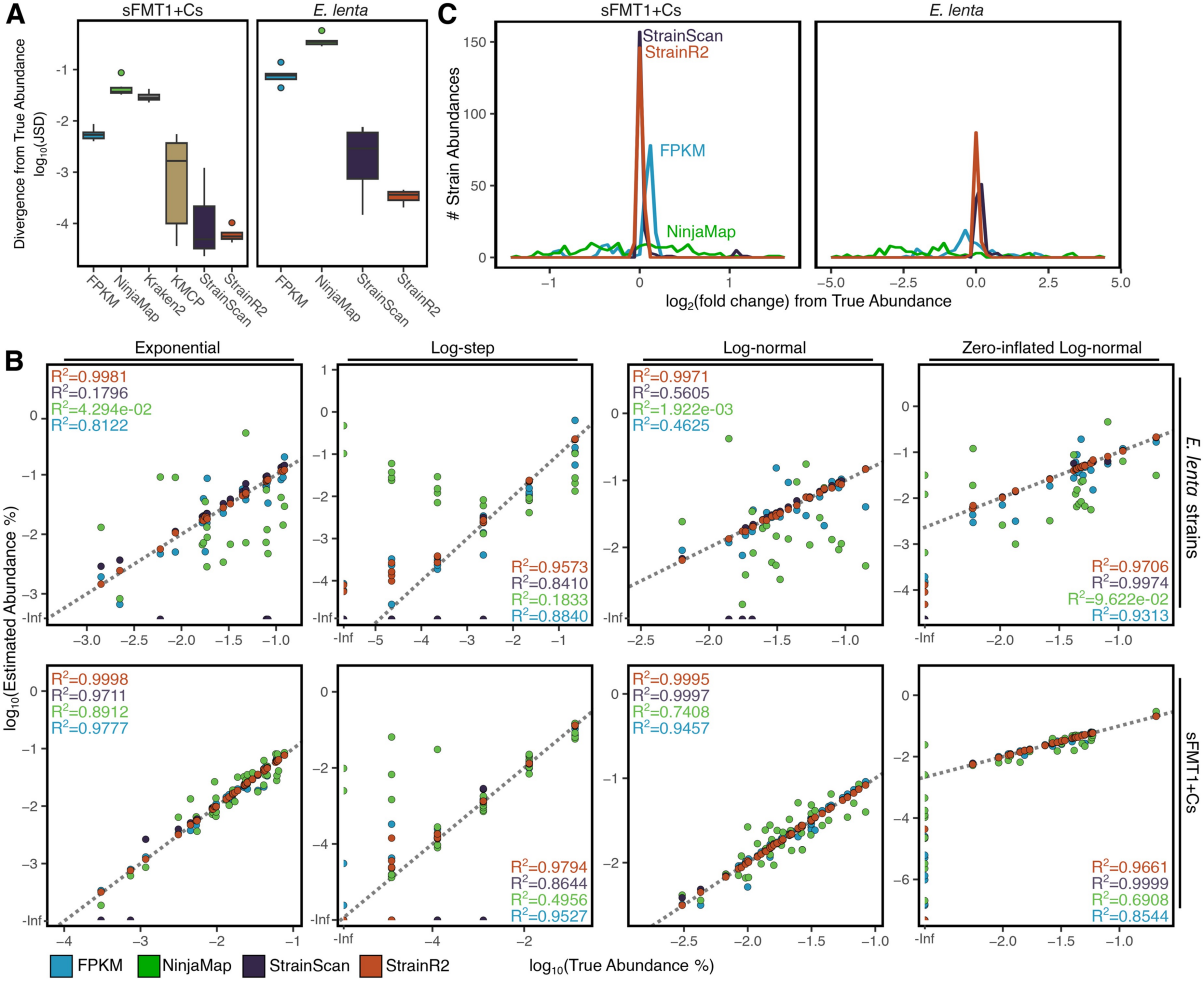
StrainR2 provides accurate strain abundances across varied community compositions. (A) Jensen–Shannon divergence between true community composition and estimated abundance are minimized by StrainR2 compared to other methods. Reads were generated *in silico* across multiple community compositions and distributions (Fig. 2, available as supplementary data at *Bioinformatics* online). (B) Scatterplots for the correlation between estimated abundances and true community compositions are shown for all mock community distributions in sFMT1+Cs and *E. lenta* strains. Inset values represent the Pearson correlation for each tool based on log_10_ transformed data with values of -Inf substituted for the minimum finite value minus 1. The uniform and missing distributions are omitted as most or all strains have the same true abundance and render the scatterplots non-informative. (C) A frequency plot of the fold-change from the true abundance shows that StrainR2 rarely predicts an abundance far from the true abundance. Data shown is the sum of all six mock community distributions both for sFMT1+Cs and *E. lenta* strains.

With uniformly abundant *E. lenta* reads, StrainR2’s wpFUKM best followed a uniform distribution out of all methods tested, as determined by coefficients of variation (Fig. 3A, available as supplementary data at *Bioinformatics* online). While median FUKM (mFUKM) is the only abundance estimate provided by StrainR1, StrainR2 is still able to quantitatively improve on this measure, with the coefficient of variation decreasing from 6.22% to 5.53%. Specifically, StrainR2 achieves this by including overlaps between subcontigs, using larger k-mers, and marking k-mers from excluded subcontigs as non-unique. Furthermore, NinjaMap showed the worst performance out of all the methods tested, with several cases of strain abundances being off by more than ten-fold (Fig. 3B, available as supplementary data at *Bioinformatics* online).

To further assess the accuracy, we examined the recovery of strain abundances across 6 different distributions (Fig. 2 and Tables 3 and 4, available as supplementary data at *Bioinformatics* online). Jensen–Shannon divergence (JSD) between true and predicted abundances was used. All measures of abundance were first converted to be a percentage of the total abundance so that all measures of abundance were comparable, and then the JSD was calculated. Resulting values can be between 0 and 1, where 0 represents the least divergence. Across all types of distributions, StrainR2 had a JSD at least two magnitudes smaller than either NinjaMap or FPKM (Fig. 3A). StrainR2 and StrainScan have similar median JSDs for the sFMT1+Cs community, though StrainR2 is more consistent, displaying lower variation between replicates. In the *E. lenta* community, StrainR2 outperforms StrainScan. Shortcomings of using JSD are that (i) it does not include cases where strains have a true or predicted abundance of zero, and (ii) low-abundance strains have a smaller impact on JSD.

Relative abundance versus true abundance for each tool in each distribution and community reveals their strengths and weaknesses (Fig. 3B). Pearson correlations were determined using the log_10_ relative abundance, with abundances of zero, or -Infinity in the log space being substituted with the lowest log_10_ abundance minus 1. StrainScan appears to have the best performance in cases where there are no low-abundance strains, as evidenced by the zero-inflated log-normal community. In cases of low-abundance strains, StrainScan tended to predict an abundance of 0, which led to low Pearson correlation, as evidenced by the “log-step” distribution. StrainScan’s inability to recover strain presence for low-abundance strains was exaggerated for the *E. lenta* community, as evidenced by the exponential and log-normal distributions.

Log_2_-fold-changes paint a more detailed picture of the abundance predictions that each tool provides (Fig. 3C). While StrainScan has a larger frequency around 0 for the sFMT1+Cs community, there are also log_2_-fold-changes >1. These values originate from low-abundance strains in the log-step community, which have a coverage around 1.66%. For these cases, StrainScan overestimated the abundance. StrainR2 does not have any cases of large abundance prediction inaccuracies and maintains the highest accuracy for the *E. lenta* community.

**Figure 4.**
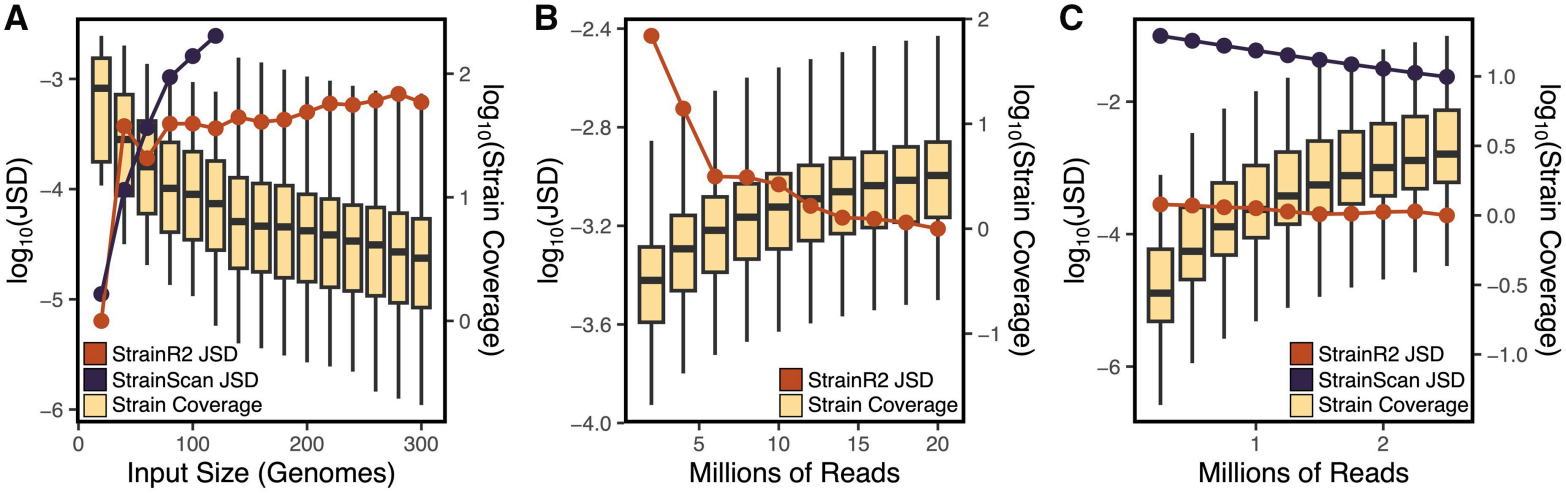
StrainR2 scales and maintains accuracy on larger inputs and smaller read depths. Accuracy, as measured through Jensen–Shannon Divergence from the true abundance, is maintained through: (A) various community sizes at 20 million reads, (B) various read depths for a 300-membered community, and (C) various read depths for the sFMT1+Cs community. StrainScan was unable to operate on the 300-membered community due to excessive memory needs and as such is not plotted in B. Coverage is also shown to demonstrate the effect of varying community size or read depth. When generating reads, the coverages for strains were drawn from a log-normal distribution.

StrainR2 can also be used to test the presence or absence of a strain depending on whether it outputs zero as an abundance. The rate of false positives and negatives heavily depends on which weighted percentile is used as the final abundance (Fig. 4A, available as supplementary data at *Bioinformatics* online). Using a higher weighted percentile increases false positives, whereas lower weighted percentiles increase false negatives, usually in the case of low-abundance organisms. Based on our analyses, a weighted percentile of 60 was chosen to compare StrainR2’s strain presence or absence predictive ability, and this value was selected as the default parameter. F1 scores of StrainR2 and four other tools reveal that StrainR2 predicted presence most reliably (Fig. 4B, available as supplementary data at *Bioinformatics* online). Only three distributions are shown, as these are the only distributions that contained absent strains. StrainScan had a low F1 score for the log-Step community due to its inability to predict the presence of low-abundance strains. These results also suggest that StrainR2 performs slightly better than YACHT for predicting the presence or absence of strains with recommended parameters and shows a large improvement over NinjaMap and FPKM. To further test the presence/absence prediction of these tools, the tools were run on ten replicates of the zero-inflated log-normal community, each with different strain abundances (Fig. 4C, available as supplementary data at *Bioinformatics* online).

**Figure 5.**
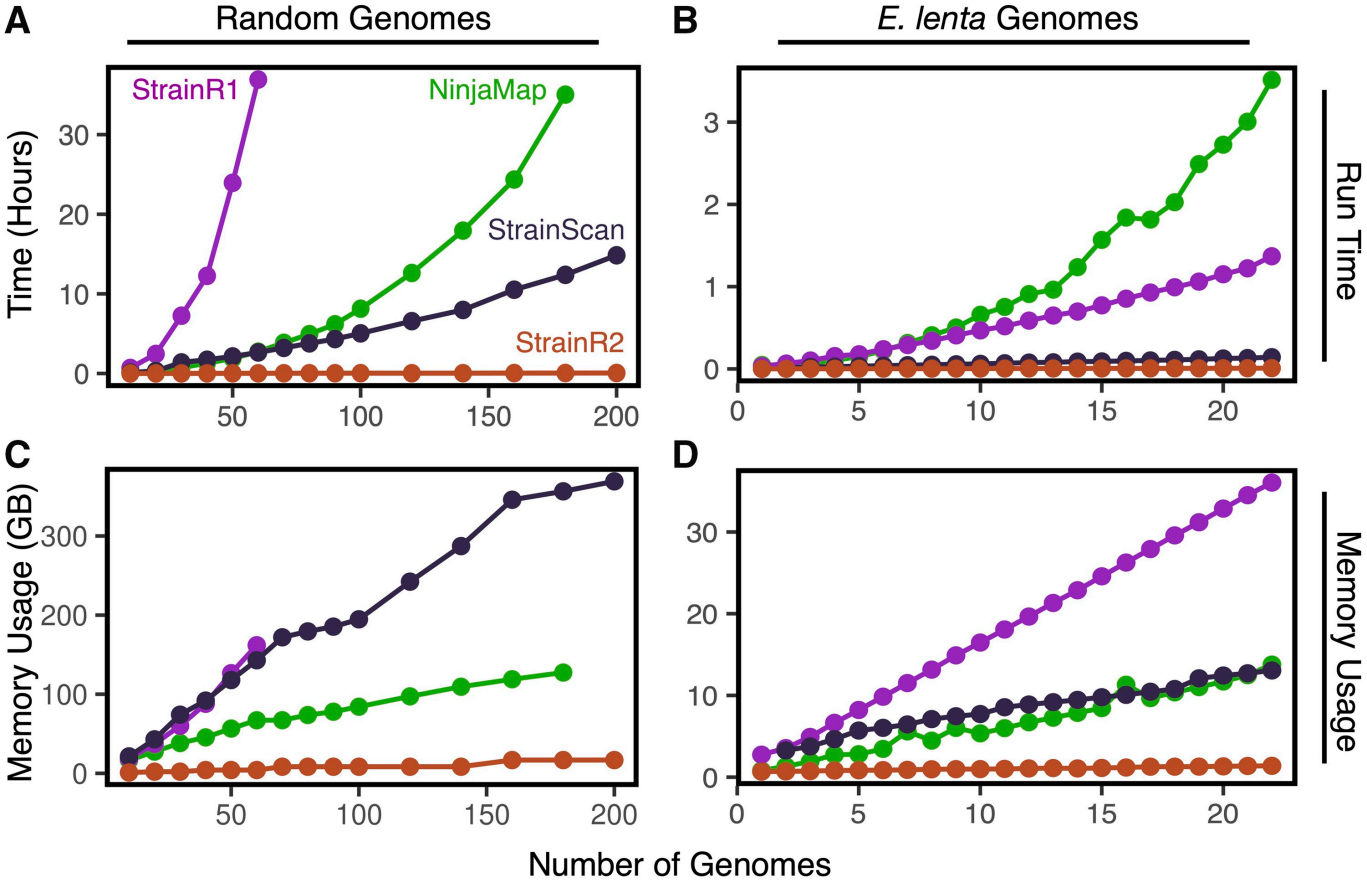
StrainR2 uses fewer system resources while scaling linearly. Run times for database generation for (A) 200 random genomes and (B) *E. lenta* strains show StrainR2 following a linear growth. StrainR2’s final run times were 3 min and 50 s, and 32 s, respectively, at maximum community complexity for random genomes and *E. lenta*, respectively. Memory usage for (C) the 200 genome input and (D) the *E. lenta* community again shows StrainR2 using the least resources, with final memory usages of 16.8 and 1.4 GB, respectively.

**Figure 6.**
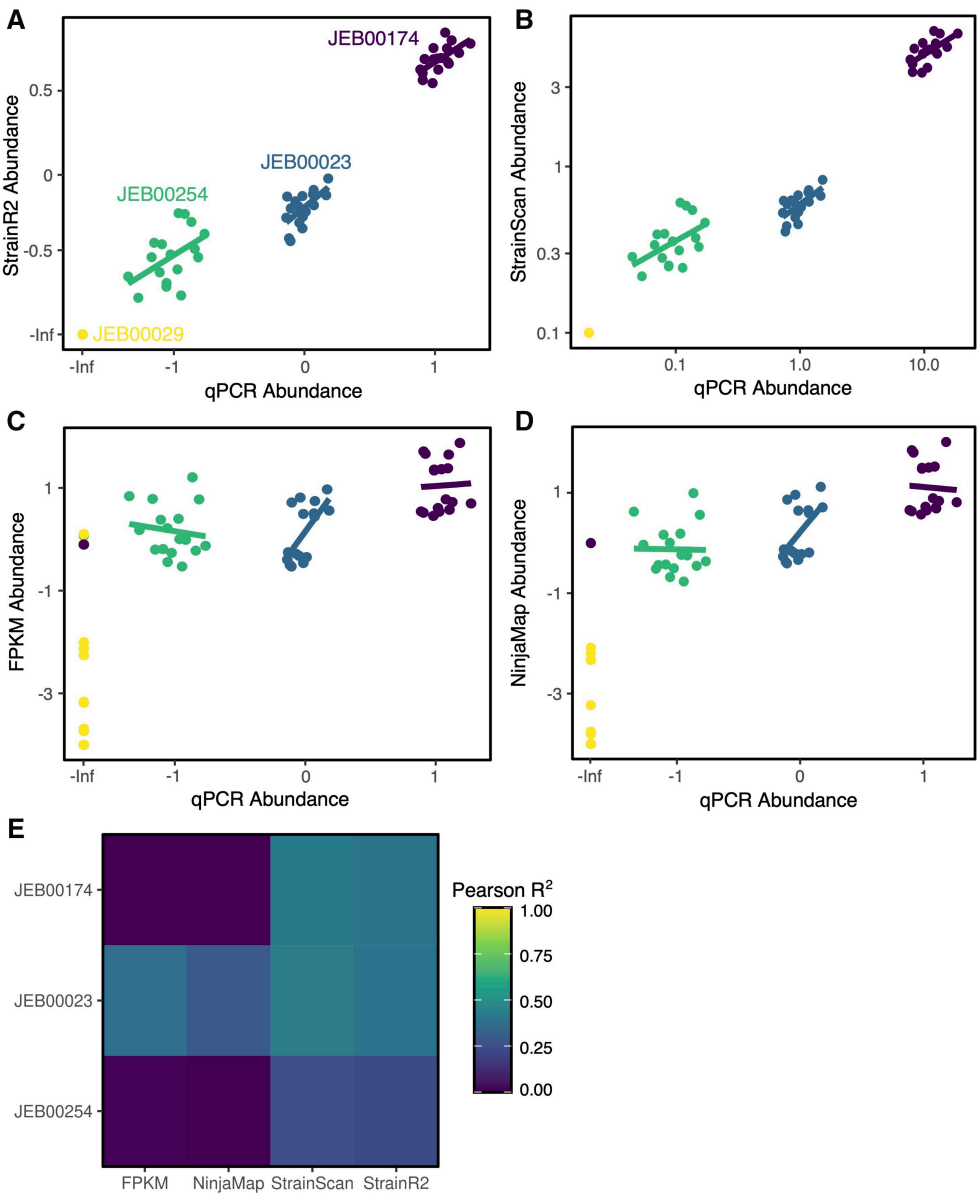
StrainR2 accurately recovers abundances measured by qPCR. Abundance predictions for qPCR as compared to (A) StrainR2, (B) StrainScan, (C) FPKM, and (D) Ninjamap. All abundances are the fold-change from the geometric mean of strain abundances within a sample and are shown on a logarithmic scale (i.e. centered log ratio). (E) Pearson correlations for each strain’s abundances versus qPCR via differing tools. Pearson correlations for all samples/strains in panels A, B, C, and D are *R*^2^ = 0.9432, *R*^2^ = 0.9501, *R*^2^ = 0.3559, and *R*^2^ = 0.3139, respectively. Each point represents the quantification of a single strain in a single animal with linear regressions drawn on a per-strain basis.

To test the limits of StrainR2 for large communities, we selected 300 genomes from an in-house collection of lab strains and genomes, filtering for strains where at least 2% of its 301-mers are unique relative to all other strains to exclude potential clonal isolates. From these genomes, there were 9 distinct phyla, 95 genera, and 170 species (Fig. 5A, available as supplementary data at *Bioinformatics* online). StrainR2 demonstrated an ability to maintain high accuracy for this community and all subsets thereof (Fig. 4A). We attempted to run StrainScan on all of the inputs, but the read mapping step was unable to provide abundance when the number of genomes exceeded 120. When running on the 120 genome input, StrainScan also falsely marked the absence of 4 of the strains, whereas StrainR2 correctly reported an abundance for every strain up to 300. When taking the same 300 strains and instead decreasing the read depth, StrainR2 maintained similar accuracy above 4 million reads (Fig. 4B), at which point the median strain coverage was 0.644 and the lowest coverage was 0.0417. Due to excessive memory usage, StrainScan was unable to create a custom database for the 300-member community, though based on the results on smaller input sizes, it also would have been unlikely to run the mapping step. To demonstrate how StrainR2 can maintain accuracy even at lower depths for typical synthetic community sizes, and to be able to compare it to StrainScan, we also tested the effect of read depth on accuracy for the 38 strain of sFMT1+Cs (Fig. 4C). Despite running StrainScan with low coverage mode on, StrainR2 showed consistently better accuracy than StrainScan, having an average of 219 times smaller JSD. This analysis also demonstrates that low-read coverage can accurately recover community composition when most strains are highly divergent. Moreover, we examined StrainR2 accuracy when using a range of read sizes as small as 50 bp paired-end reads and observed relatively stable accuracy, although increasing read length increases the probability of the read covering an informative variant base (Fig. 6, available as supplementary data at *Bioinformatics* online).

To assess how StrainR2 scales with synthetic community complexity, the memory in GB and run times of the computationally intensive database generation steps of StrainR2, StrainR1, NinjaMap, and StrainScan were gathered as described in the implementation section on an Ubuntu server with dual Intel Xeon Silver 4214 CPUs and 384 GB of memory (Fig. 5). Input sizes up to 200 genomes were tested (Fig. 5B, available as supplementary data at *Bioinformatics* online). Run times for StrainR2 grew linearly and remained low for all inputs as compared to StrainR1 (Fig. 5A), and the memory usage showed similar trends (Fig. 5C). StrainScan presented high memory needs for larger inputs. To test if the trend would hold for highly similar inputs, runs were performed on one through 22 of the *E. lenta* strains. StrainR2 maintained its low run times, whereas the other tool’s run times grew non-linearly (Fig. 5B). Memory usage on the *E. lenta* strains is also shown (Fig. 5D). StrainR2 run times scale closely with the number of unique k-mers in a community, meaning it is unaffected by highly similar communities (Fig. 7, available as supplementary data at *Bioinformatics* online). To validate function outside of a high-performance computing environment, the sFMT1+Cs community was profiled through a StrainR2 Bioconda installation on a personal computer. The system was running OS X Ventura 13.1 with an Apple M1 Pro processor and 16 GB of RAM. The run times were 1 min 31 s and 6 min 56 s for PreProcessR and StrainR, respectively, for 51.9 million reads (7.8 Gbases) of paired-end NovaSeq 6000 data with eight threads. This highlights that StrainR2 does not require high-performance computing nodes and is a tangible strategy available to most research groups.

While StrainR2 was shown to have the best accuracy *in silico*, we sought to validate its function using experimental samples and a gold-standard method for strain quantification: qPCR with strain-specific probes. Shotgun metagenomic sequencing data were obtained from 17 fecal samples of gnotobiotic mice colonized with sFMT1 or sFMT1+Cs. As a control, two of the mice were also germ-free. To determine the true abundance of strains, qPCR was performed on samples from the same mice for four of the strains present in sFMT1 (JEB00023, JEB00029, JEB00174, and JEB00254). JEB00023 and JEB00174 were selected as they are both strains of the species *Bacteroides uniformis* and represent an important use case of StrainR2. JEB00029 was selected as our previous experiments had suggested it could not colonize the mice, while JEB00254 was capable of colonization at low abundances (Tian *et al.* 2025). To allow for direct comparison of relative abundances from metagenomics to absolute abundances from qPCR, data were normalized as the fold-change from the geometric mean of strain abundances within a sample (centered log ratio).

StrainR2 and StrainScan maintained a close relationship with the results obtained from qPCR, as well as correctly predicting when strains were absent, as was the case with JEB00029 and the two germ-free mice (Fig. 6A and B). NinjaMap showed a weaker correlation with the data from qPCR and was inconsistent with predicting abundances of strains between samples (Fig. 6D), with FPKM having similar results (Fig. 6C). FPKM and NinjaMap both incorrectly assigned abundances to JEB00029 and strains in the germ-free mice, showing that another strength of StrainR2 is more accurate presence/absence prediction, as it was the only tool to agree with qPCR on the absence of strains. Per-strain correlations with each tool are described in Fig. 6E. Pearson correlation values for StrainR2, NinjaMap, FPKM, and StrainScan versus copies/g across all strains are *R*^2^ = 0.9432, *R*^2^ = 0.3139, *R*^2^ = 0.3559, and *R*^2^ = 0.9501, respectively.

## 4 Conclusions

Through analysis of both *in silico* and experimental data, we demonstrate that StrainR2 provides highly accurate strain abundances and prevalences using a fraction of the computational resources of previous approaches. StrainR2 makes shotgun metagenomic sequencing reads a viable tool for accurate strain abundances in synthetic communities without the need for high-performance computing. This may eliminate the need for more time-consuming or expensive methods to assess strain abundance, such as qPCR, to which it yields comparable abundances. StrainR2 is also able to provide abundances in scenarios where designing primers for qPCR would be extremely difficult or impossible, as would be the case for strain-competition experiments where strains are highly similar. StrainR2 is available via GitHub, Bioconda, and as a Docker container.

## Supplementary Material

btaf440_Supplementary_Data

## Data Availability

Source code and required resources to regenerate *in silico* reads are available via GitHub. Sequencing data are available via the NCBI Sequence read archive under BioProject PRJNA1038784.
