# Supplementary material for: StrainR2 accurately deconvolutes strain-level abundances in synthetic microbial communities
Source: Bioinformatics. 2025 Aug 6;41(8):btaf440. doi: 10.1093/bioinformatics/btaf440 (PMC12377904; doi:10.1093/bioinformatics/btaf440)
Supplement: btaf440_Supplementary_Data [file btaf440_supplementary_data.zip › Heber_StrainR2_Supplement.pdf]

StrainR2 accurately deconvolutes strain-level abundances in synthetic microbial communities

Kerim Heber[1], Shuchang Tian[1], Daniela Betancurt-Anzola[1], Heejung Koo[1], Jordan E. Bisanz[1,2]
[1]Department of Biochemistry and Molecular Biology, Pennsylvania State University, University Park, PA 16802, USA
[2]One Health Microbiome Center, Huck Life Sciences Institute, University Park, PA 16802, USA

Supplemental Materials

Figures S1-S7
Tables S1-S4

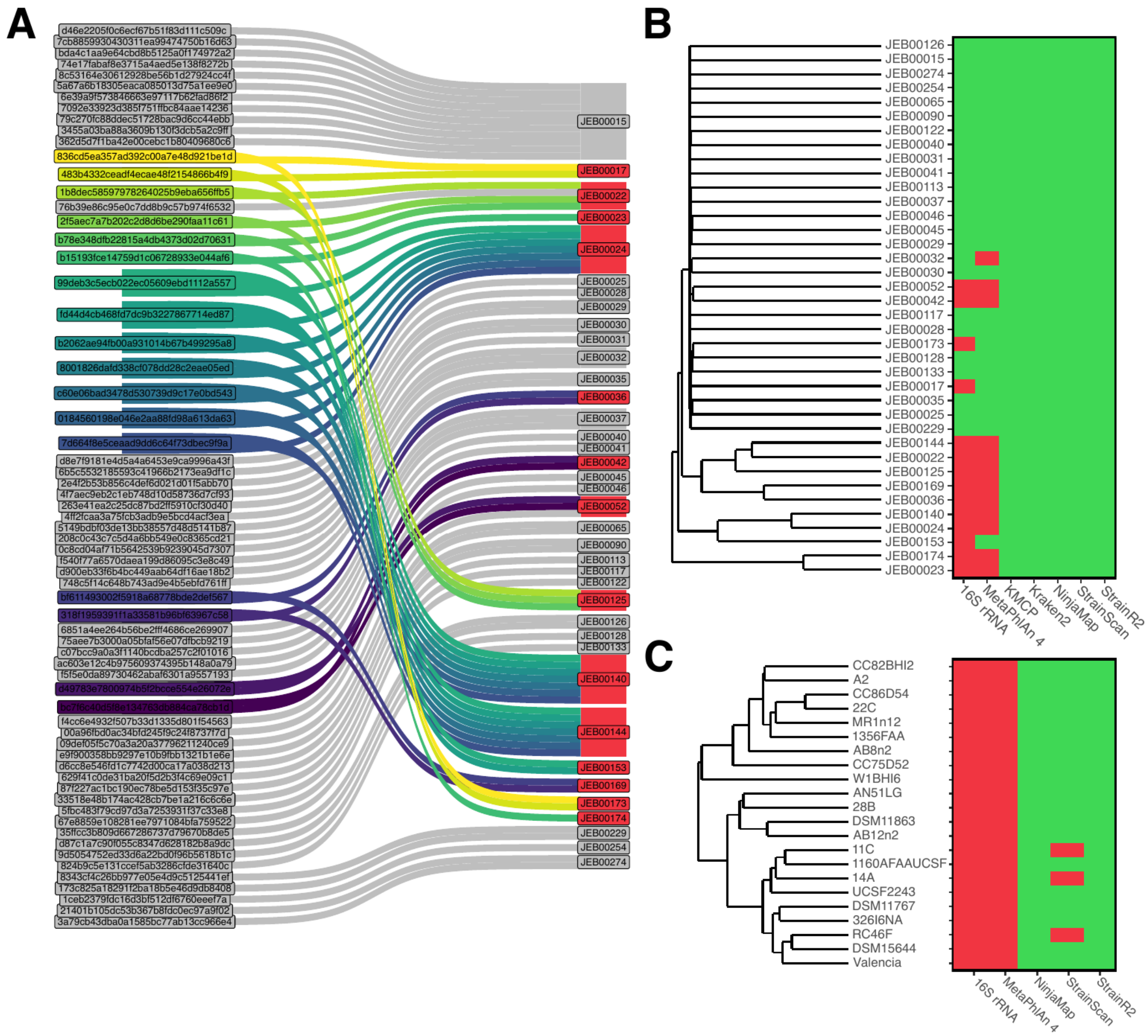

Figure S1. Conventional methods fail to resolve strain-level abundances in synthetic communities. (A) In a 38 strain synthetic community, amplicon sequencing variants (ASVs) for the V4 region of the 16S rRNA gene (left) may map to multiple strains (right), making abundance resolution impossible. Gray ASVs and strains indicate unique mapping and strains colored red have ambiguous mapping. Heatmaps show which strains are uniquely (green) and ambiguously (red) resolved for 16S rRNA sequencing and various methods that utilize shotgun metagenomic sequencing for the **(B)** 38 strain sFMT1+Cs and **(C)** 22 strain *E. lenta* communities. KMCP and Kraken2 are excluded from analysis for **(C)** due to an inability to assign NCBI taxonomy IDs and not running as a result.

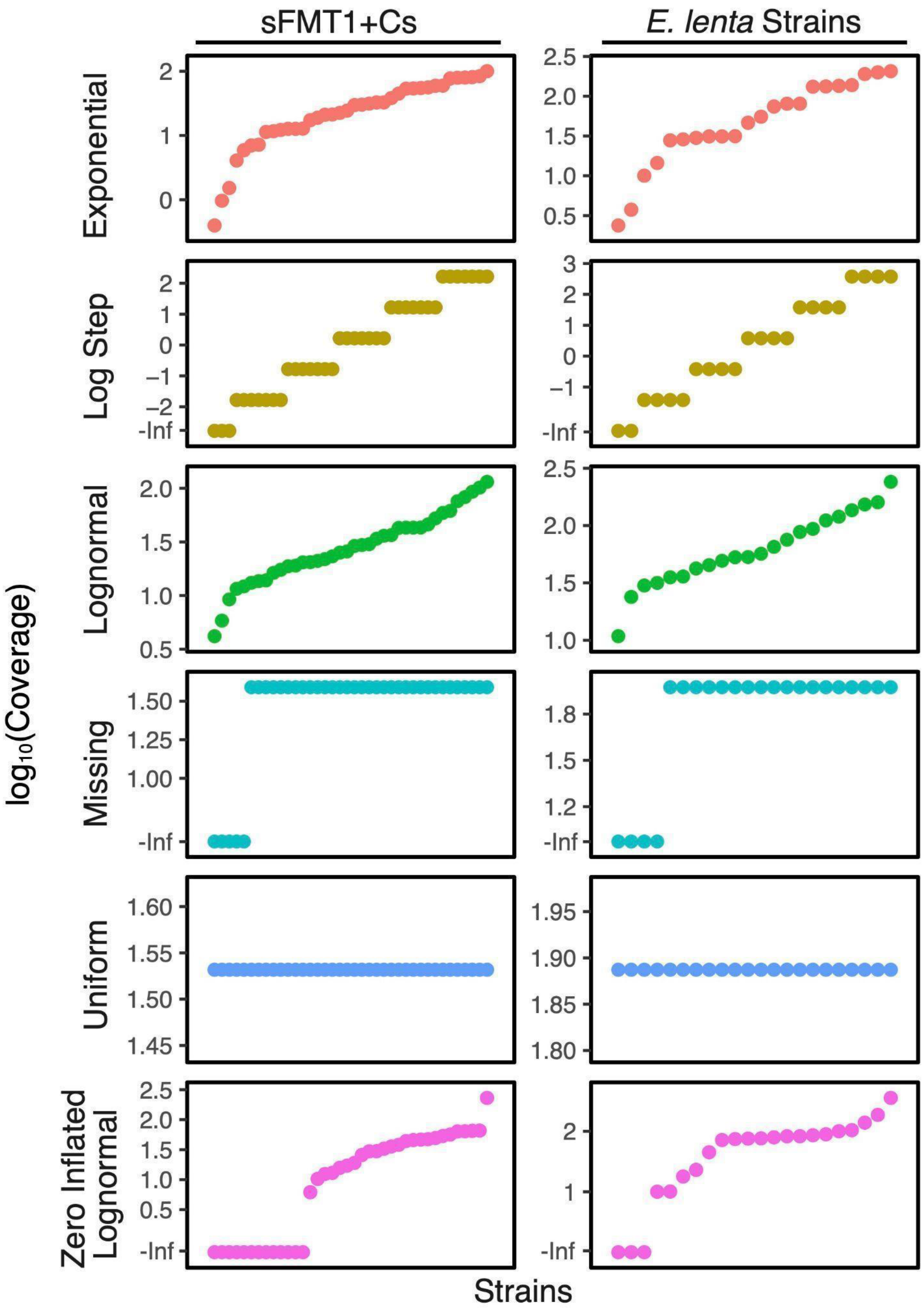

Figure S2. Mock reads simulate a variety of distributions. The 6 predefined *in silico* read distributions were designed to test the performance of tools in predicting presence/absence and scenarios where strains had low abundances or were not present. Abundances are defined as the read coverage depth of each strain. Strain coverages are reported in **Tables S3 and S4.**

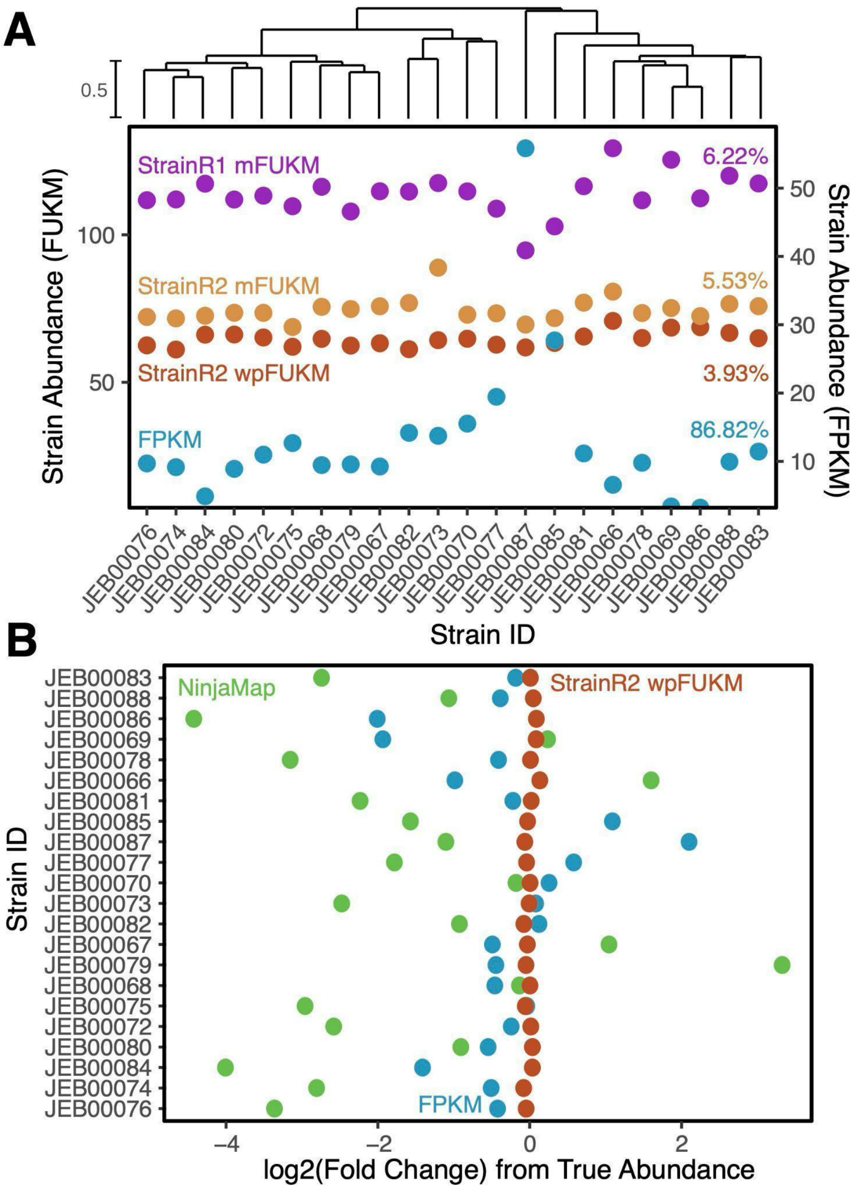

Figure S3. StrainR2 improves upon abundance estimation of *E. lenta* strains when compared to StrainR1. (A) Estimated abundances for a uniformly abundant community of *E. lenta* strains shows that StrainR2's wpFUKM most closely follows a uniform distribution. Percentages shown to the right indicate coefficient of variation. **(B)** Accuracy as measured by $\log_2$(fold-change) demonstrates that StrainR2 is able to correct abundances to a high degree of accuracy, whereas NinjaMap and FPKM perform with much lower accuracy.

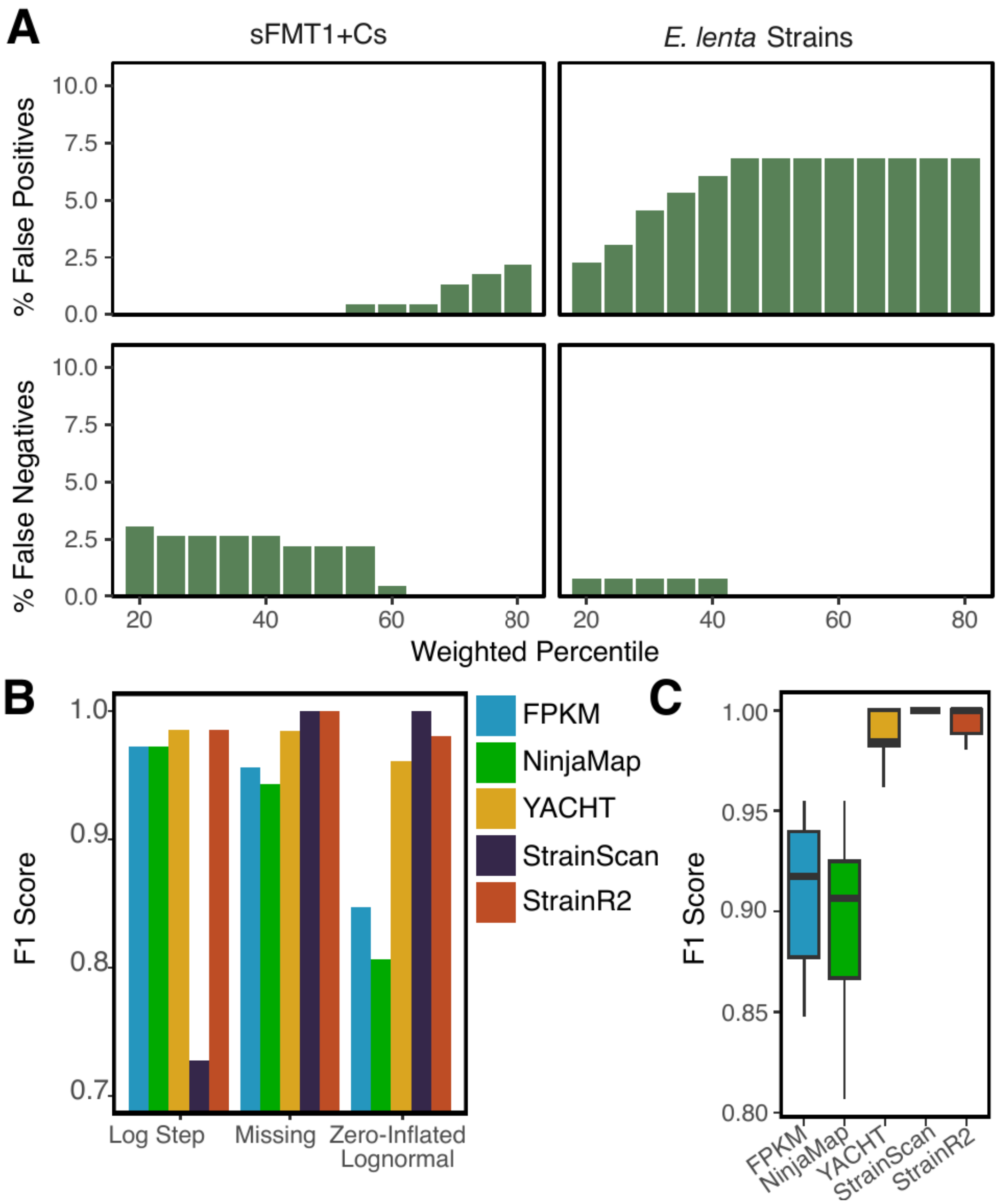

Figure S4. StrainR2 predicts presence or absence of strains with improved accuracy compared to other tools. (A) The average percentage of false positives or negatives across the 6 read distributions of sFMT1+Cs and *E. lenta* across different weighted percentiles of FUKM. **(B)** StrainR2 has the highest F1 scores across 3 of the read distributions and for the four tools. **(C)** F1 score for ten replicates of the zero-inflated log-normal distribution.

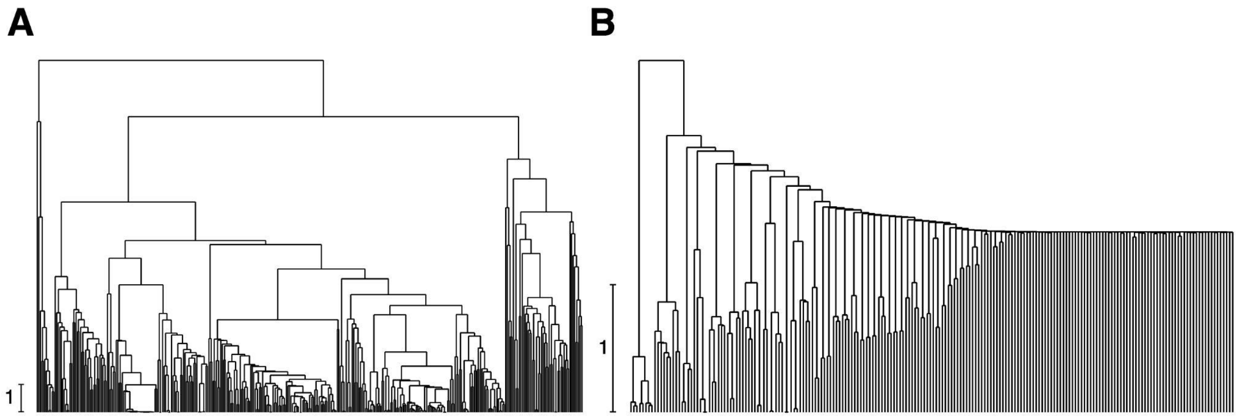

Figure S5. Dendrograms for larger communities. (A) The 300 genomes used in scalability analysis for **Figure 4** and **(B)** The 200 genomes used in resource usage testing for **Figure 5**.

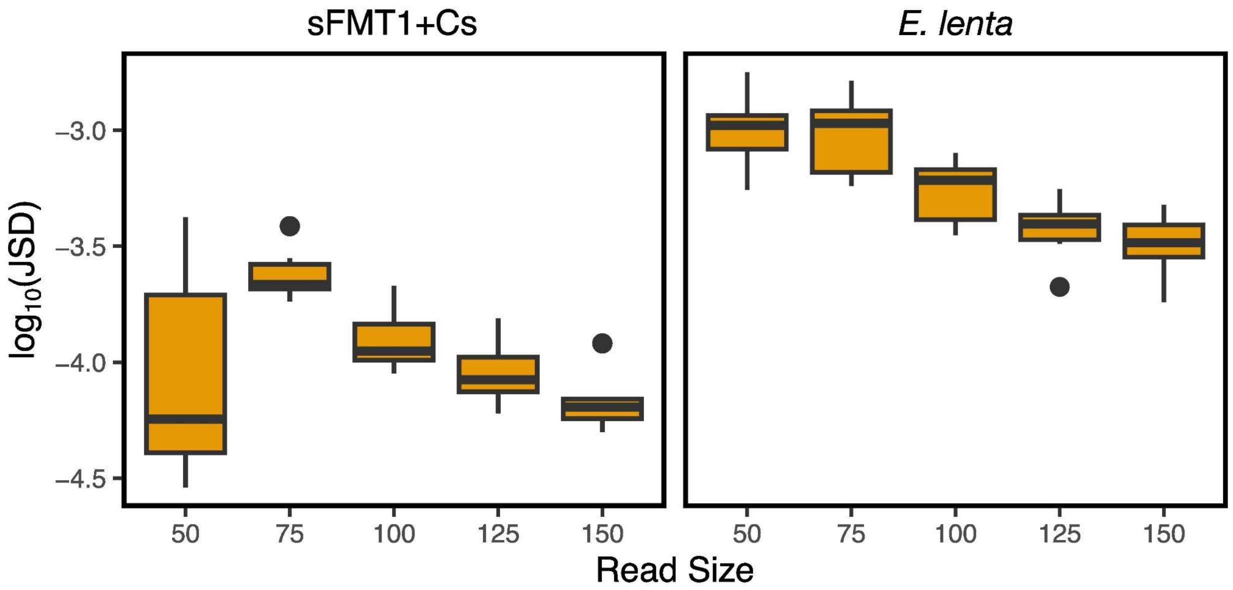

Figure S6 Accuracy of abundance prediction remains stable through various read sizes. 150 base pair paired end reads were generated from the six synthetic community distributions, then trimmed to various sizes. StrainR2's accuracy at predicting abundance remained relatively similar through various read sizes.

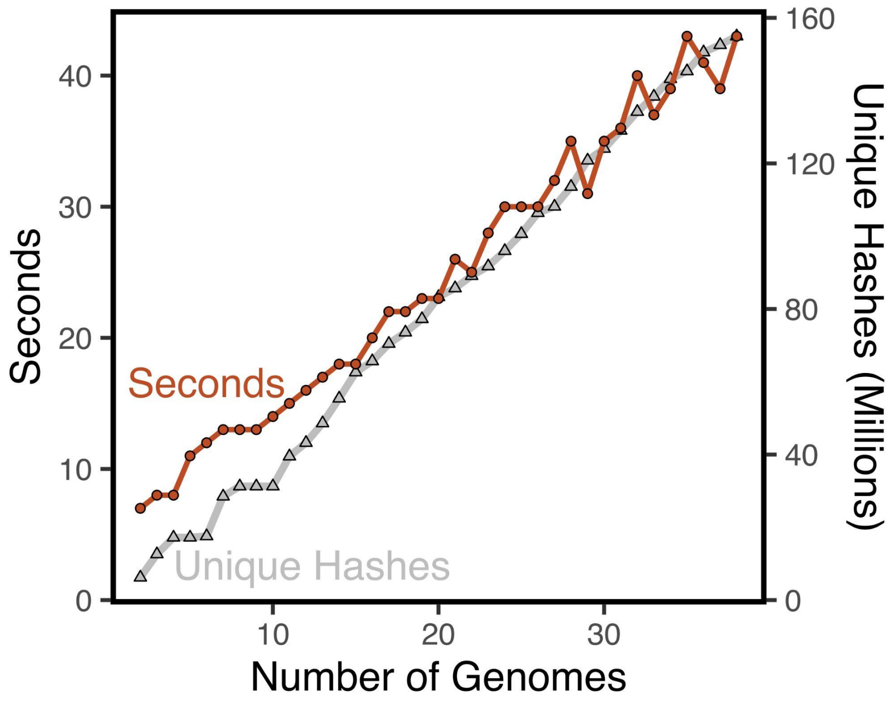

Figure S7. StrainR2 run times scale closely with the number of unique hashes in the input. Run times are plotted on varying input sizes from genomes in the sFMT1+Cs community. Total unique hash counts are shown, which estimate how many unique k-mers there are in the inputted genomes. Run times scale closely with unique hashes, meaning the more similar a community is, the better the run time will scale.

Table S1. Genome accessions for sFMT strains

LabID	Species	Strain ID	Genome Accession
JEB00015	*Escherichia coli*	DSM 18039	GCA_000005845.2
JEB00017	*Bacteroides thetaiotaomicron*	DSM 2079	GCA_014131755.1
JEB00022	*Bacteroides ovatus*	DSM 1896	GCA_001314995.1
JEB00023	*Bacteroides uniformis*	DSM 6597	JBEUMQ000000000
JEB00024	*Bacteroides vulgatus*	DSM 1447	GCA_000012825.1
JEB00025	*Parabacteroides merdae*	DSM 19495	GCA_900445495.1
JEB00028	*Enterocloster asparagiformis*	DSM 15981	GCA_000158075.1
JEB00029	*Dorea longicatena*	DSM 13814	GCA_000154065.1
JEB00030	*Agathobacter rectalis*	DSM 17629	GCA_000209935.1
JEB00031	*Clostridium scindens*	DSM 5676	GCA_004295125.1
JEB00032	*Lachnospira eligens*	DSM 3376	GCA_000146185.1
JEB00035	*Bacteroides stercoris*	DSM 19555	GCA_900106605.1
JEB00036	*Bacteroides xylanisolvens*	DSM 18836	GCA_000210075.1
JEB00037	*Anaerobutyricum hallii*	DSM 3353	GCA_000173975.1
JEB00040	*Lactonifactor longoviformis*	DSM 17459	GCA_002915525.1
JEB00041	*Faecalibacterium prausnitzii*	DSM 17677	GCA_010509575.1
JEB00042	*Blautia producta*	DSM 3507	GCA_002915535.1
JEB00045	*Dorea formicigenerans*	DSM 3992	GCA_000169235.1
JEB00046	*Blautia obeum*	DSM 25238	GCA_000153905.1
JEB00052	*Blautia producta*	DSM 2950	GCA_010669205.1
JEB00065	*Clostridium spiroforme*	DSM 1552	GCA_000154805.1
JEB00090	*Eggerthella lenta*	DSM 2243	GCA_003339945.1
JEB00113	*Eubacterium hadrus*	DSM 3319	GCA_000332875.2
JEB00117	*Clostridium orbiscindens*	NCBI 1_3_50AFAAA	GCA_000760655.1
JEB00122	*Clostridium symbiosum*	NCBI WAL-14673	GCA_000189615.1
JEB00125	*Bacteroides ovatus*	NCBI D2	GCA_000159075.2
JEB00126	*Bifidobacterium longum*	NCBI 35B	GCA_000261225.1
JEB00128	*Bacteroides caccae*	NCBI CL03T12C61	GCA_000273725.1
JEB00133	*Bacteroides cellulosilyticus*	NCBI CL02T12C19	GCA_000273015.1
JEB00140	*Bacteroides vulgatus*	NCBI CL09T03C04	GCA_000273295.1
JEB00144	*Bacteroides ovatus*	NCBI 3_8_47FAA	GCA_000218325.1
JEB00153	*Bacteroides dorei*	NCBI CL03T12C01	GCA_000273075.1
JEB00169	*Bacteroides xylanisolvens*	2_1_22	GCA_000162155.1
JEB00173	*Bacteroides finegoldii*	NCBI CL09T03C10	GCA_000304195.1
JEB00174	*Bacteroides uniformis*	4_1_36	GCA_000185585.1
JEB00229	*Parabacteroides sp.*	D13	GCA_000162275.1
JEB00254	*Peptostreptococcus anaerobius*	CC14N	SAMN42012254
JEB00274	*Sutterella wadsworthensis*	NCBI HGA0223	GCA_000411515.1

Table S2. Genome accessions for *E. lenta* strains

Lab ID	Strain	GenBank Accession
JEB00066	Eggerthella lenta 1356FAA	GCA_000185625.1
JEB00067	Eggerthella lenta 11C	GCA_003340245.1
JEB00068	Eggerthella lenta 14A	GCA_003340255.1
JEB00069	Eggerthella lenta 22C	GCA_003340195.1
JEB00070	Eggerthella lenta 28B	GCA_003340165.1
JEB00072	Eggerthella lenta DSM11767	GCA_003340045.1
JEB00073	Eggerthella lenta DSM11863	GCA_003340015.1
JEB00074	Eggerthella lenta DSM15644	GCA_003340005.1
JEB00075	Eggerthella lenta UCSF2243	GCA_003339975.1
JEB00076	Eggerthella lenta Valencia	GCA_003339885.1
JEB00077	Eggerthella lenta AN51LG	GCA_003340155.1
JEB00078	Eggerthella lenta MR1n12	GCA_003339935.1
JEB00079	Eggerthella lenta 1160AFAAUCSF	GCA_003340395.1
JEB00080	Eggerthella lenta 326I6NA	GCA_003340465.1
JEB00081	Eggerthella lenta AB8n2	GCA_003340145.1
JEB00082	Eggerthella lenta AB12n2	GCA_003340445.1
JEB00083	Eggerthella lenta CC82BHI2	GCA_003340075.1
JEB00084	Eggerthella lenta RC46F	GCA_003339915.1
JEB00085	Eggerthella lenta CC75D52	GCA_003340405.1
JEB00086	Eggerthella lenta CC86D54	GCA_003340065.1
JEB00087	Eggerthella lenta W1BHI6	GCA_003339875.1
JEB00088	Eggerthella lenta A2	GCA_003340125.1

Table S3. Strain coverages in mock sFMT distributions

StrainID	Exponential	Log step	Log-normal	Missing Strain	Zero inflated log-normal	Uniform
JEB00015	32.73	0.00	9.21	39.00	63.99	34.03
JEB00017	4.09	0.00	29.03	0.00	0.00	34.03
JEB00022	5.90	0.00	33.94	39.00	29.96	34.03
JEB00023	54.00	0.02	13.66	39.00	0.00	34.03
JEB00024	12.21	0.02	46.10	39.00	17.24	34.03
JEB00025	30.24	0.02	4.19	39.00	66.11	34.03
JEB00028	77.30	0.02	16.30	0.00	38.21	34.03
JEB00029	12.71	0.02	42.78	39.00	0.00	34.03
JEB00030	0.97	0.02	17.43	39.00	0.00	34.03
JEB00031	11.37	0.02	36.12	39.00	0.00	34.03
JEB00032	83.98	0.17	13.88	39.00	0.00	34.03
JEB00035	11.69	0.17	25.15	39.00	65.34	34.03
JEB00036	32.79	0.17	20.43	39.00	44.11	34.03
JEB00037	1.53	0.17	92.80	39.00	0.00	34.03
JEB00040	54.97	0.17	19.03	0.00	19.09	34.03
JEB00041	22.48	0.17	52.49	39.00	64.35	34.03
JEB00042	81.15	0.17	12.20	39.00	49.57	34.03
JEB00045	18.94	1.66	58.98	39.00	0.00	34.03
JEB00046	56.17	1.66	11.58	39.00	10.34	34.03
JEB00052	12.71	1.66	61.60	39.00	46.74	34.03
JEB00065	53.66	1.66	21.90	39.00	0.00	34.03
JEB00090	12.80	1.66	20.52	0.00	12.49	34.03
JEB00113	0.40	1.66	30.21	39.00	32.90	34.03
JEB00117	17.33	1.66	75.89	39.00	0.00	34.03
JEB00122	59.77	16.61	29.59	39.00	13.04	34.03
JEB00125	29.75	16.61	43.12	39.00	29.71	34.03
JEB00126	80.07	16.61	82.83	39.00	15.78	34.03
JEB00128	38.29	16.61	23.25	39.00	47.56	34.03
JEB00133	24.44	16.61	18.76	39.00	231.90	34.03
JEB00140	59.55	16.61	5.84	39.00	0.00	34.03
JEB00144	7.02	16.61	43.00	39.00	0.00	34.03
JEB00153	21.05	166.12	21.10	39.00	25.83	34.03
JEB00169	21.26	166.12	43.07	0.00	35.83	34.03
JEB00173	100.42	166.12	101.61	39.00	6.19	34.03
JEB00174	44.96	166.12	36.63	39.00	56.75	34.03
JEB00229	31.39	166.12	25.81	39.00	0.00	34.03
JEB00254	79.39	166.12	114.89	39.00	45.92	34.03
JEB00274	7.20	166.12	13.17	39.00	53.74	34.03

Table S4. Strain coverages in mock *E. lenta* distributions

StrainID	Exponential	Log step	Log-normal	Missing Strain	Zero inflated log-normal	Uniform
JEB00079	80.48	0.00	23.89	94.28	139.19	77.14
JEB00067	10.06	0.00	75.27	94.28	71.80	77.14
JEB00066	14.50	0.04	88.01	0.00	10.12	77.14
JEB00068	132.77	0.04	35.42	94.28	0.00	77.14
JEB00069	30.02	0.04	119.53	94.28	104.47	77.14
JEB00070	74.35	0.04	10.86	0.00	186.78	77.14
JEB00080	190.05	0.38	42.27	94.28	86.06	77.14
JEB00088	31.25	0.38	110.91	94.28	10.06	77.14
JEB00082	2.38	0.38	45.20	94.28	0.00	77.14
JEB00081	27.95	0.38	93.66	94.28	357.17	77.14
JEB00077	206.48	3.82	35.98	94.28	74.48	77.14
JEB00085	28.74	3.82	65.21	94.28	76.55	77.14
JEB00083	80.62	3.82	52.97	0.00	76.00	77.14
JEB00086	3.75	3.82	240.63	94.28	23.11	77.14
JEB00072	135.15	38.18	49.35	94.28	79.58	77.14
JEB00073	55.29	38.18	136.11	94.28	82.98	77.14
JEB00074	199.54	38.18	31.63	0.00	17.91	77.14
JEB00078	46.56	38.18	152.92	94.28	45.08	77.14
JEB00084	138.10	381.83	30.02	94.28	0.00	77.14
JEB00075	31.25	381.83	159.73	94.28	89.21	77.14
JEB00076	131.93	381.83	56.79	94.28	100.41	77.14
JEB00087	31.48	381.83	53.20	94.28	82.90	77.14
